# Supplementary material for: Variation in human mobility and its impact on the risk of future COVID-19 outbreaks in Taiwan
Source: BMC Public Health. 2021 Jan 27;21:226. doi: 10.1186/s12889-021-10260-7 (PMC7838857; doi:10.1186/s12889-021-10260-7)
Supplement: Supplementary file 1 — Additional file 1. Supplementary materials. [file 12889_2021_10260_MOESM1_ESM.pdf]

SUPPLEMENTARY MATERIALS

SUPPLEMENTARY TEXT

Estimating P_{ij}

We built a travel model to estimate the proportion of time people living in location *i* spend in location *j* (P_{ij}) by fitting the model to the Facebook movement data. X_{ij} represents the proportion of people living in location *i* currently in location *j*, and $\widehat{X_{ij}}$ represents the equilibrium state of X_{ij}, and its value under the fitted model is used as our estimate of P_{ij}. People living in location *i* travel with probability F_i, and the probability that a traveler from location *i* travels to location *j* is denoted by T_{ij}. Travelers go back to their home location at probability λ_i per unit of time. $M_{ij,t,t+1}$ represents the number of people moving from location *i* to location *j* between time *t* and *t*+1.

$$X_{ij}(t+1) = X_{ij}(t) + X_{ii}(t)F_iT_{ij} - X_{ij}(t)\lambda_i$$
$$X_{ii}(t+1) = X_{ii}(t) - X_{ii}(t)F_i + \sum_{j\neq i} X_{ij}(t)\lambda_i$$
$$M_{ij,t,t+1} = N_iX_{ii}(t)F_iT_{ij} + N_jX_{ji}(t)\lambda_j$$
$$M_{ii,t,t+1} = N_iX_{ii}(t)(1-F_i) + \sum_{j\neq i} N_jX_{ji}(t)\left(1-\lambda_j\right)$$

At equilibrium,

$$\widehat{X_{ij}} = \frac{F_iT_{ij}}{F_i+\lambda_i},\ \widehat{X_{ii}} = \frac{\lambda_i}{F_i+\lambda_i},\ \widehat{M_{ij}} = \frac{N_iF_iT_{ij}\lambda_i}{F_i+\lambda_i} + \frac{N_jF_jT_{ji}\lambda_j}{F_j+\lambda_j},\ \text{and}\ \widehat{M_{ii}} = \frac{N_i(1-F_i)\lambda_i}{F_i+\lambda_i} + \sum_{j\neq i}\frac{N_jF_jT_{ji}(1-\lambda_j)}{F_j+\lambda_j}.$$

For simplicity, we assumed that the majority of travel is work-related travel and on average travelers spend eight hours in the travel destination (λ_i =1 given the unit of time is 8 hours) and that T_{ij} is proportional to M_{ij}, leaving F_i the only parameters to be fitted. We used a gradient descent algorithm to find the local optimum solution for F_i, where the cost function is defined by the sum of the squared difference between normalized m_{ij} and the normalized value of M_{ij} from the model. We calculated $\widehat{X_{ij}}$ under fitted parameters to obtain estimates of P_{ij}.

Residence model

The model shown in Methods considered both that (1) non-travelers get infected by infectious visitors to their home location (the first part in the following equation) and that (2) susceptible travelers get infected when they travel (the second part in the following equation).

$$\frac{dS_i}{dt} = -S_iP_{ii}\frac{R_0}{D_I}\frac{\sum_{j\ includes\ i} I_jP_{ji}}{\sum_{j\ includes\ i} N_jP_{ji}} - S_i\sum_{j\neq i} P_{ij}\frac{I_j}{N_j}\frac{R_0}{D_I}$$

Because it is possible that visitors from different cities interact inside another third city, to address how this influences the model outcome, we constructed another model where infected individuals in the city susceptible travelers travel to include infected visitors from other cities.

$$\frac{dS_i}{dt} = -S_i P_{ii} \frac{R_{0_i}}{D_I} \left(\frac{I_i P_{ii} + \sum_{j \neq i} I_j P_{ji}}{N_i P_{ii} + \sum_{j \neq i} N_j P_{ji}} \right) - S_i \sum_{j \neq i} P_{ij} \left(\frac{I_i P_{ii} + \sum_{j \neq i} I_j P_{ji}}{N_i P_{ii} + \sum_{j \neq i} N_j P_{ji}} \right) \left(\frac{R_{0_j}}{D_I} \right)$$

Because the difference between models with and without considering the interaction occurring between visitors from different cities inside another third one were minimal (Figure S11), we reported results from the simpler model in this study.

SUPPLEMENTARY FIGURES

Figure S1. Movement patterns estimated from the Facebook data in Taiwan. (A) Regular movement data. (B) Colocation matrices. The maps were plotted using shape files from Taiwan Map Store.[38]

(A)

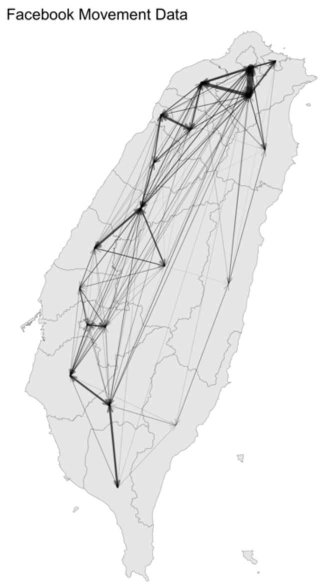

(B)

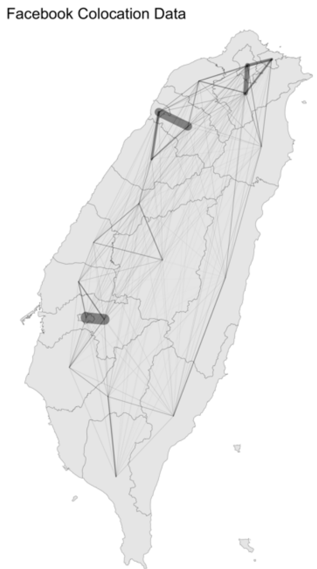

Figure S2. Mobility change over time. Two examples of city pairs where the baseline travel first decreased and then increased between February and June were shown for both **(A)** colocation and **(B)** movement data. The dates of major holidays (Lunar New Year, Ching Ming Festival, and Dragon Boat Festival) are shown in blue.

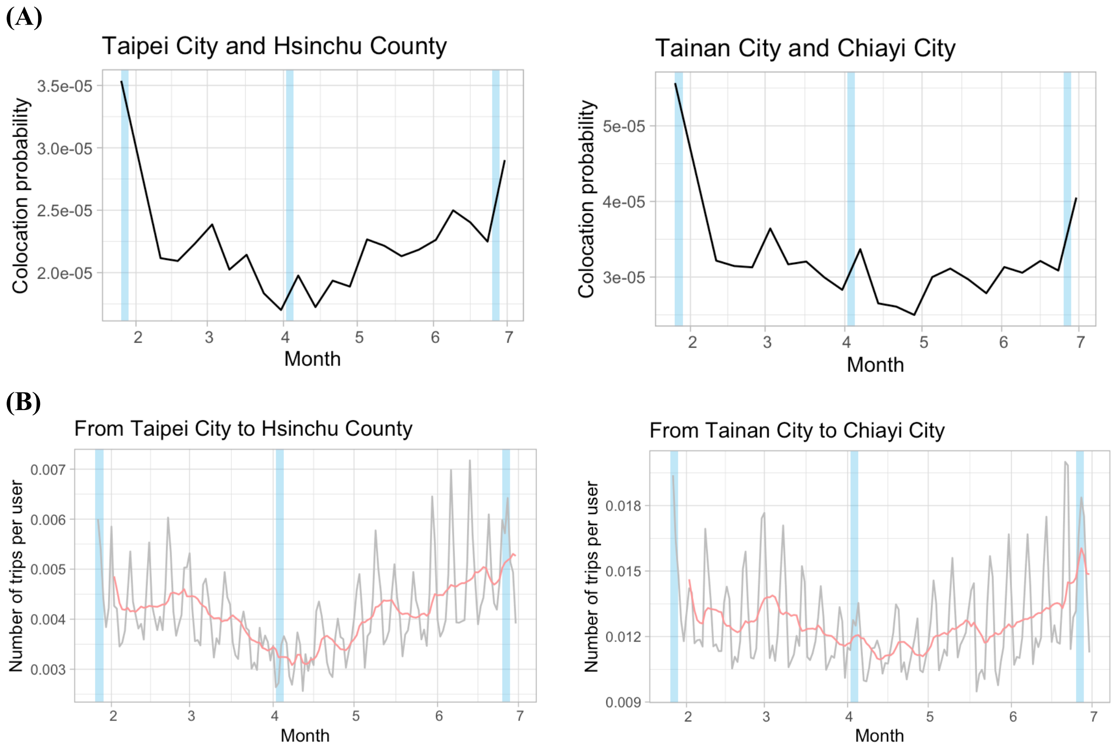

Figure S3. Cumulative number of local cases in Taiwan.

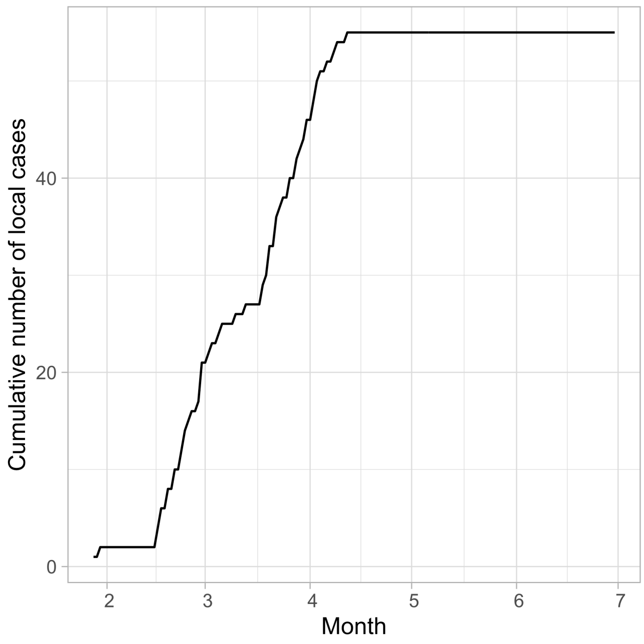

Figure S4. Disease spread was associated with measures of connectivity. In the contact model, **(A)** the probability of having more than 1000 infections (P_{1000}) increased with risk of infection (Spearman's correlation test, rho= 0.99, p-value=1.04×10^{-15}), and **(B)** the time it took to reach 1000 infections (T_{1000}) decreased with risk of infection (Spearman's correlation test, rho= -0.97, p-value=7.83×10^{-6}). **(C)** In the residence model, the variation in infection numbers across cities at T_{1000} (denoted by V_{1000}) decreased with values of source of importation (Spearman's correlation test, rho= -0.64, p-value=0.004). R_0=2.4.

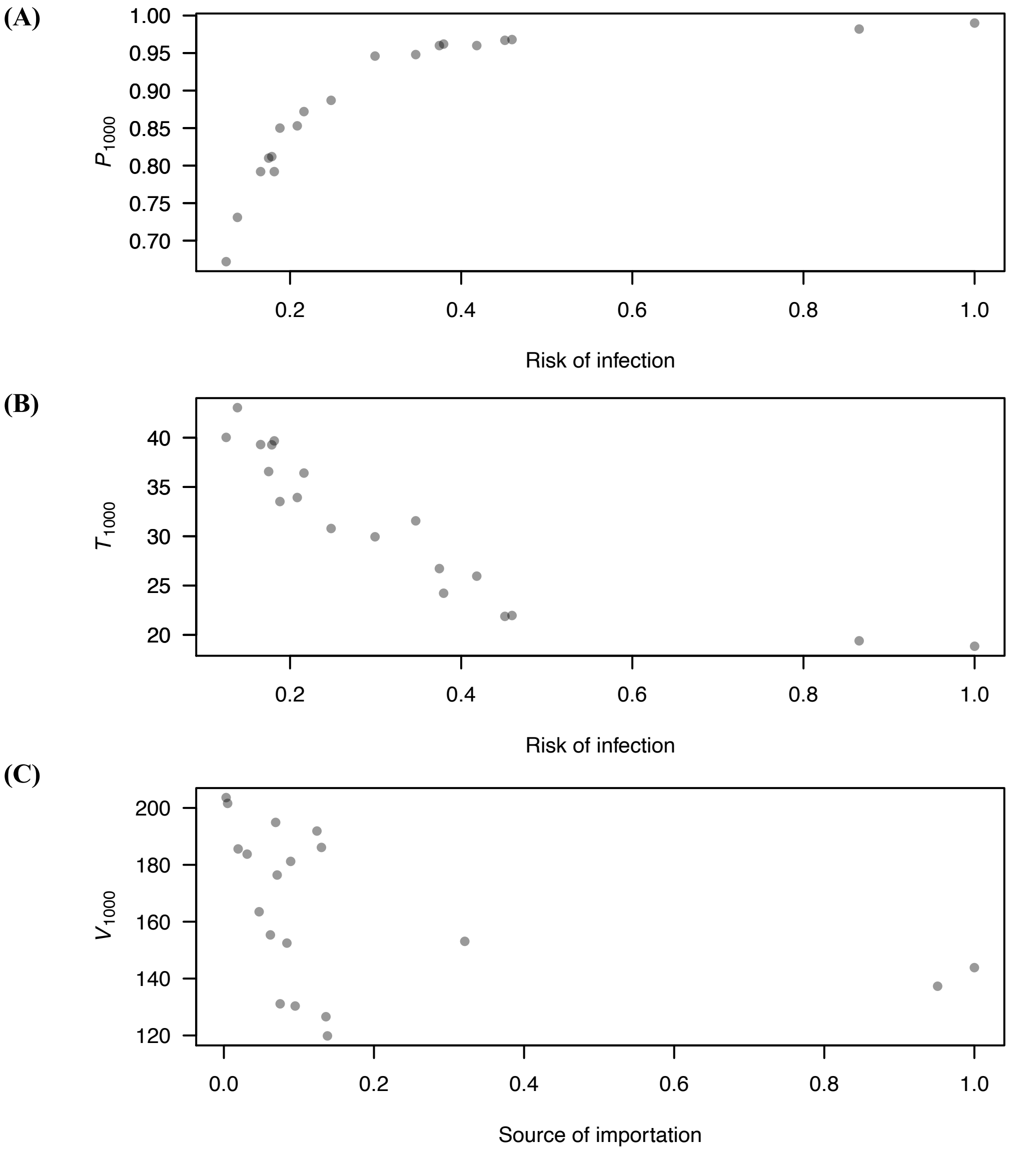

Figure S5. ***P_{1000}*** **and** ***T_{1000}*** **did not vary much with the locations of initial infections in the residence model.** R_0=2.4.

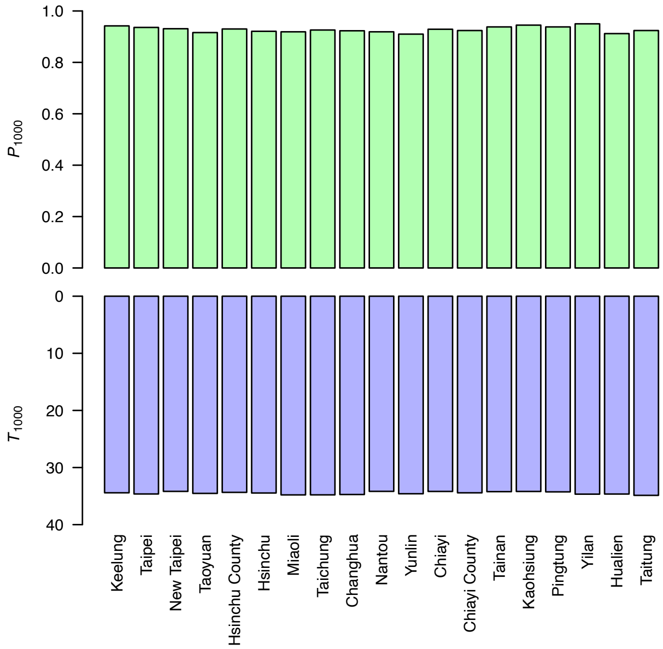

Figure S6. The impact of holiday travel on the probability of outbreak. The probability of outbreak (P_{1000}) increased with mobility during Lunar New Year in Pingtung (a smaller city). The impact of Ching Ming Festival (4-day) and Dragon Boat Festival (4-day) is less apparent. Colors represent the different timing of when initial infections occurred (blue: at the beginning of holidays; red and green: 7 days and 14-days before holidays, respectively). After holidays, mobility changed back to that during normal days and stayed the same until the end of each simulation. R_0=2.4.

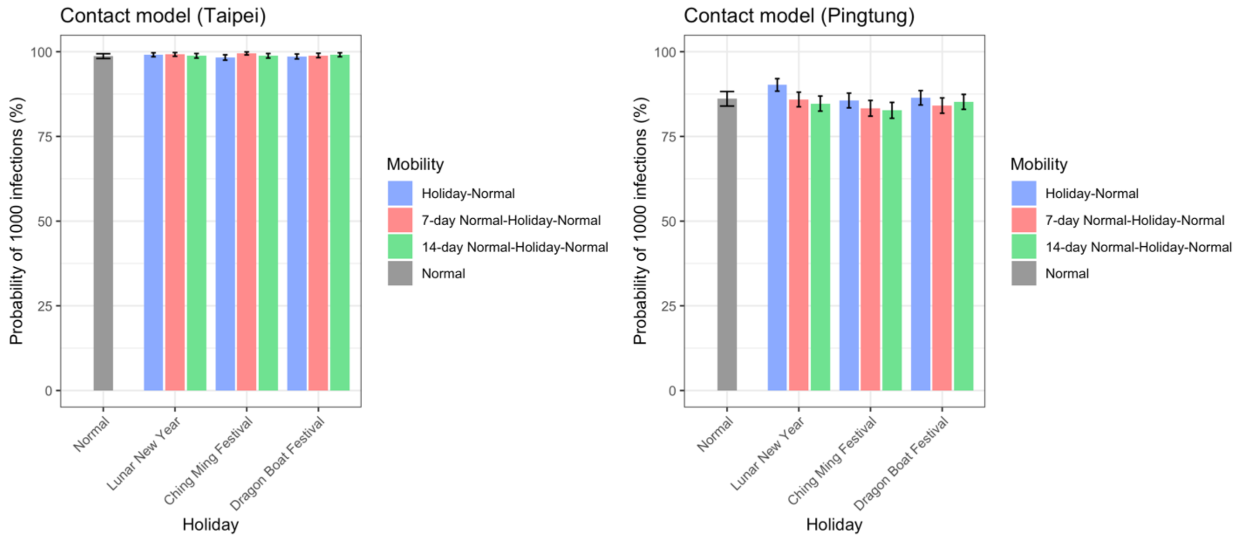

Figure S7. The impact of travel reduction on time to reach 1000 accumulated infections. If initial infections were in a big city, it took less time to reach 1000 infections in the contact model. The difference between big and small cities was not significant in the residence model. Intracity and overall travel reduction delayed the time to reach 1000 infections in both models, while intercity reduction did not. For some conditions, $P_{1000,3}$ was 0 and no bar was shown. Here travel reduction was applied during the whole time and R_0=2.4.

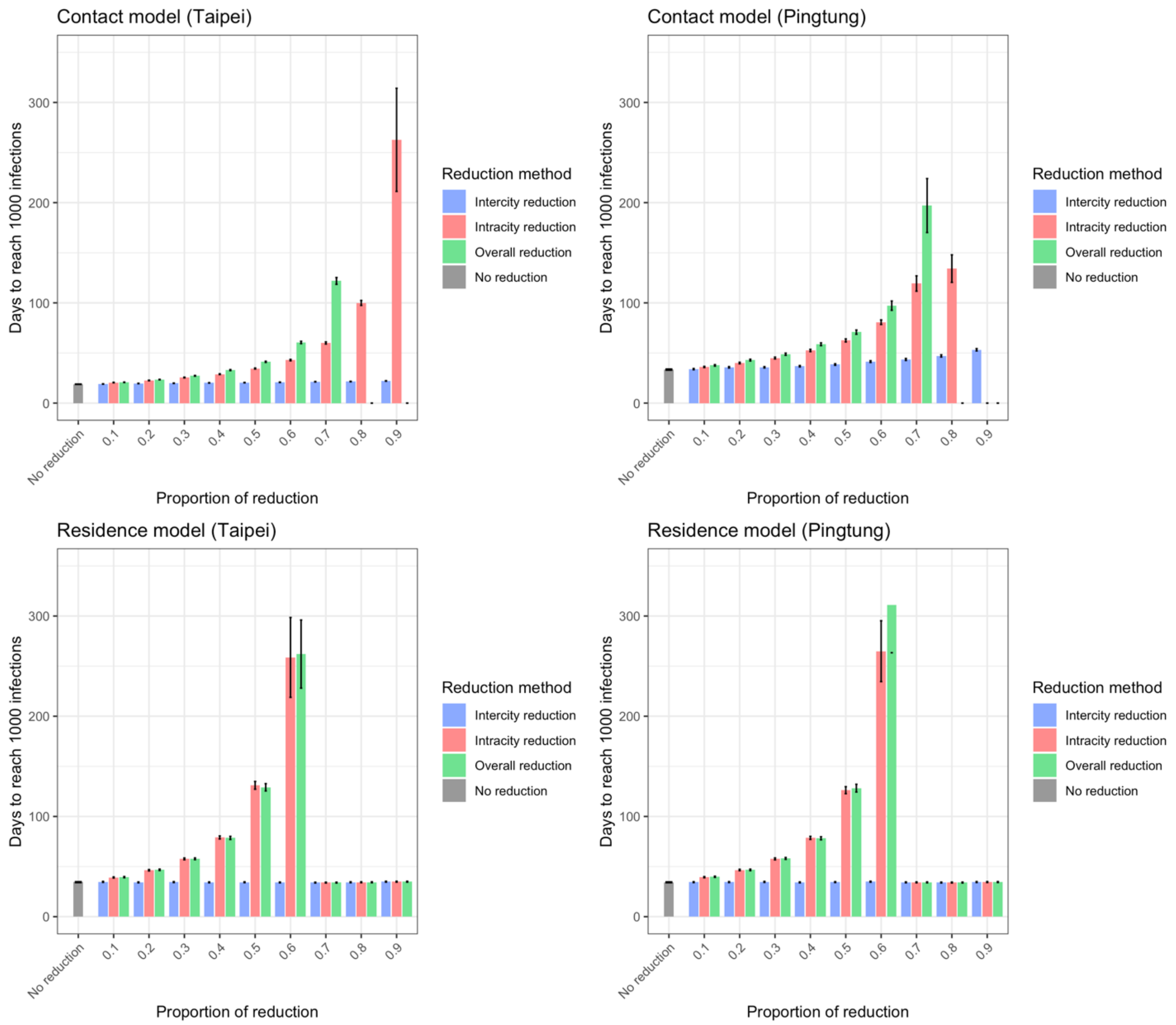

Figure S8. The impact of travel reduction on the geographic distribution of infections. Standard deviation of infection numbers across different cities when there are 1000 infections ($V_{1000,3}$) was shown. Intercity travel reduction increased the variation in infection numbers across cities in both models. Here travel reduction was applied during the whole time and R_0=2.4.

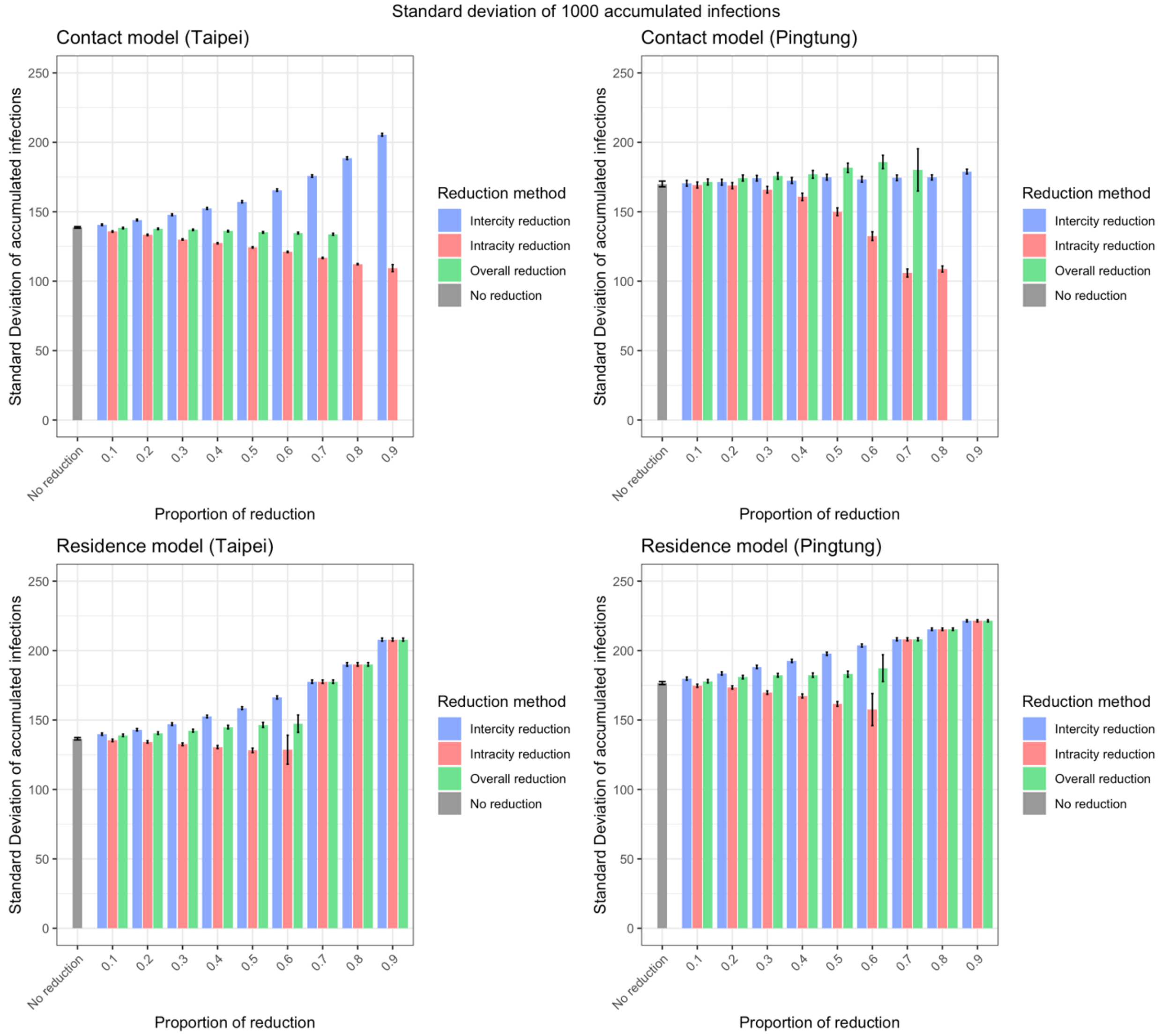

Figure S9. $T_{1000,3}$ and $V_{1000,3}$ under different lengths of intercity travel reduction. $T_{1000,3}$ (upper panel) and $V_{1000,3}$ (lower panel). Here initial infections were in Taipei city and R_0=2.4.

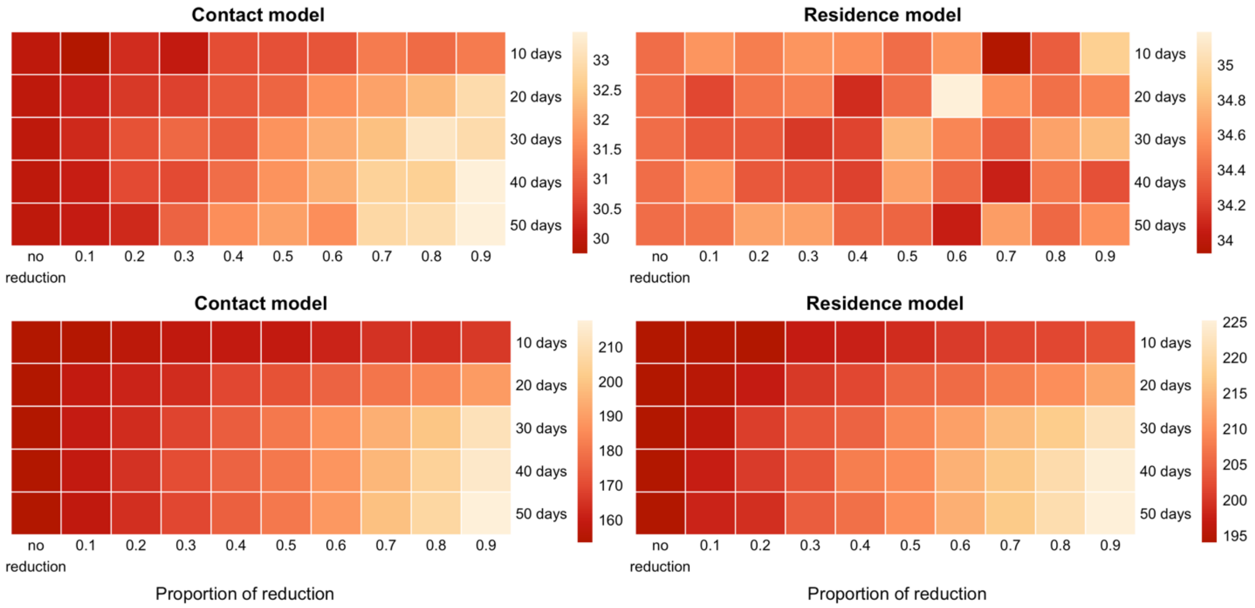

Figure S10. $P_{1000,3}$ when travel reduction started at different conditions. $P_{1000,3}$ when travel reduction started from the beginning of the simulations (denoted by 0), or when there were 10, 20, 30, 50, and 100 infections in both contact (left) and residence (right) models. Two different lengths of travel reduction duration were shown: **(A)** 10 days **(B)** 1 month. Only intracity travel reduction was shown here because intercity travel reduction only had minimal impact on $P_{1000,3}$ and the results from overall reduction and intracity reduction were qualitatively similar. It was best to reduce travel at the beginning if the duration was for 10 days or 1 month. Here initial infections were in Taipei city and R_0=2.4.

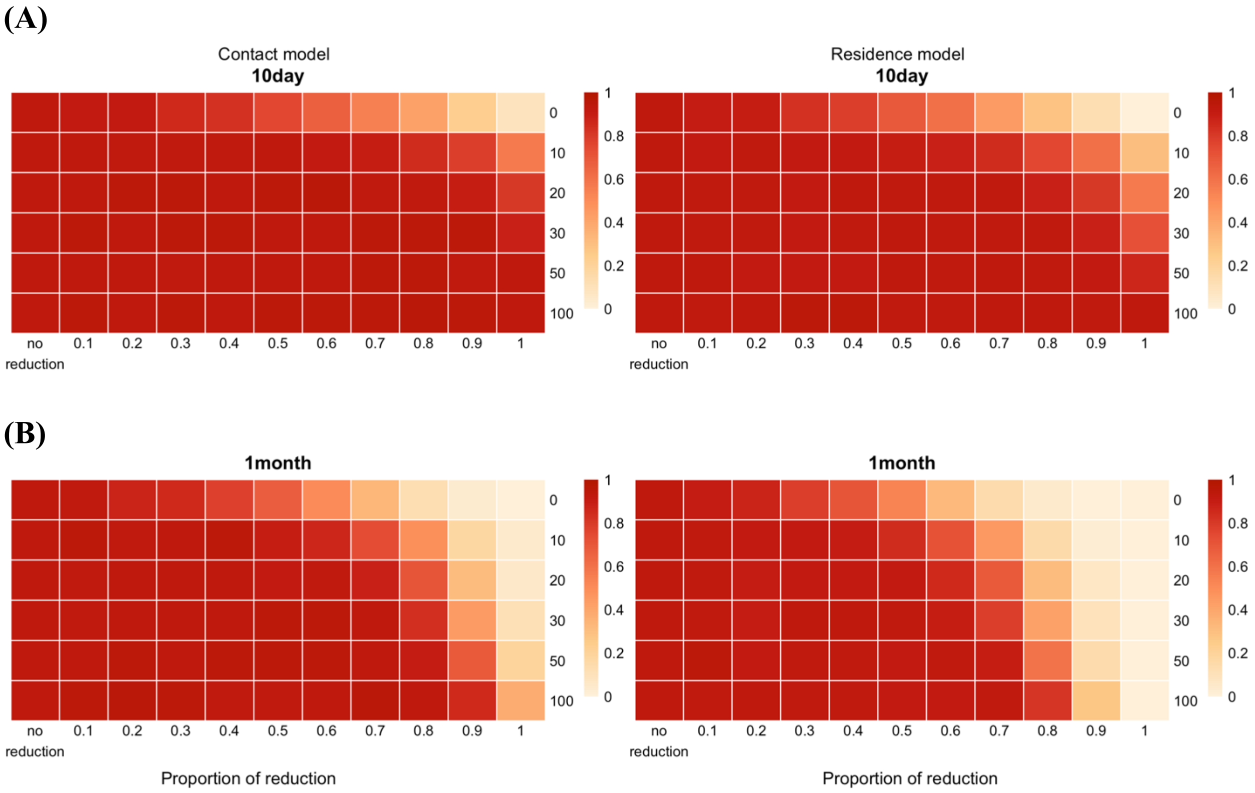

Figure S11. The comparison in $P_{1000,3}$ under two types of residence model. The results from residence models with and without considering the interaction occurring between visitors from different cities inside another third one were similar.

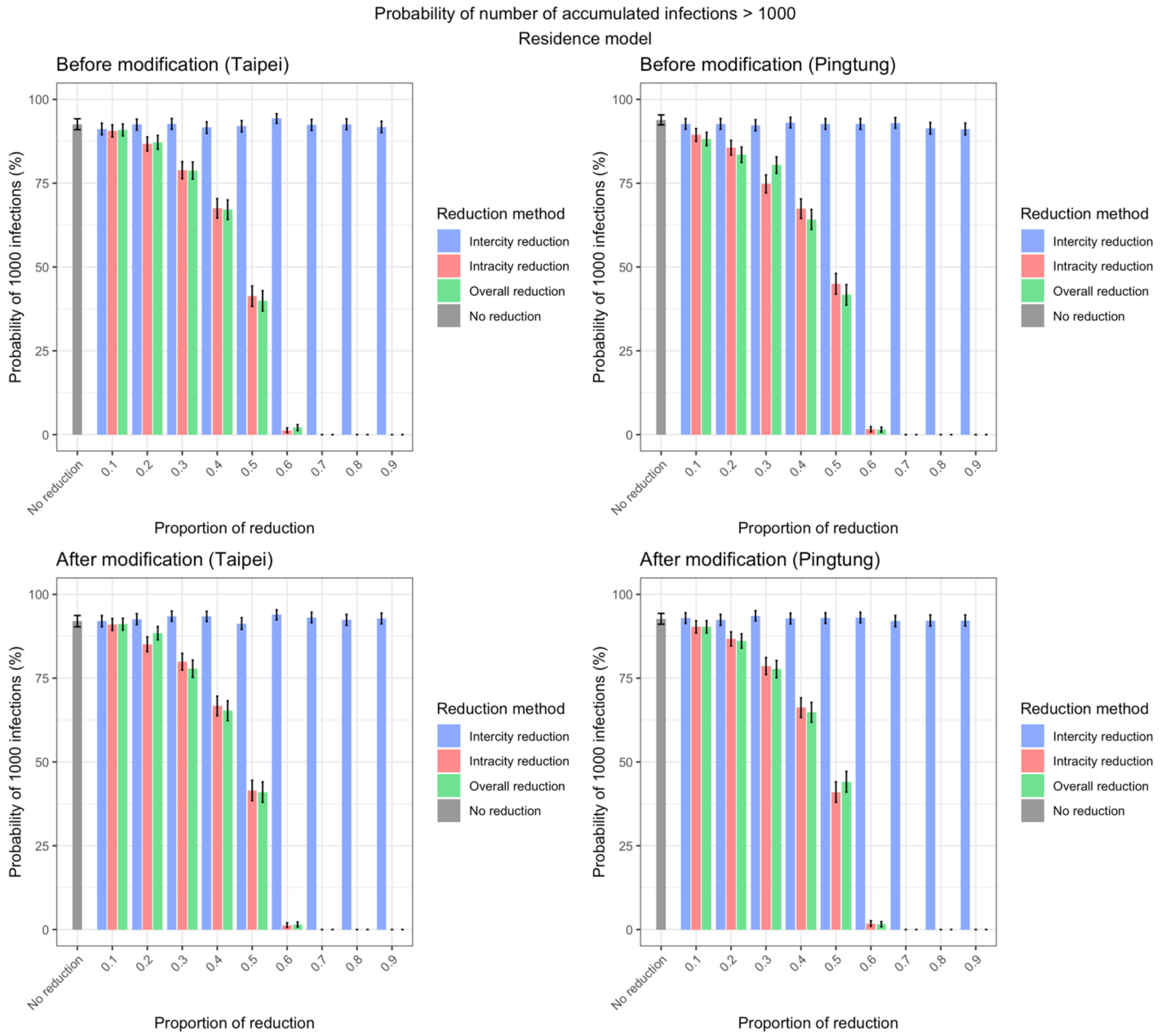

SUPPLEMENTARY TABLES

Table S1. Intracity R_0, intercity R_0, risk of infection, and risk of importation.

City	Intracity R_0	Intercity R_0	Risk of infection	Risk of importation	Source of importation
Keelung City	1.016	**0.348**	**0.451**	0.107	0.095
New Taipei City	**2.247**[#]	**0.368**	**0.865**	**0.125**	**1.000**
Taipei City	**2.500**	**0.523**	**1.000**	**0.155**	**0.951**
Taoyuan City	0.985	0.162	0.379	0.073	**0.321**
Hsinchu County	0.562	0.187	0.248	**0.123**	**0.136**
Hsinchu City	**1.023**	**0.241**	**0.418**	**0.143**	**0.138**
Miaoli County	0.425	0.104	0.175	0.044	0.062
Taichung City	**1.051**	0.081	0.375	0.026	0.130
Changhua County	0.475	0.074	0.182	0.029	0.089
Yunlin County	0.348	0.071	0.138	0.019	0.031
Chiayi County	0.258	0.121	0.125	0.073	0.084
Chiayi City	0.836	**0.212**	0.347	**0.141**	0.075
Nantou County	0.408	0.092	0.166	0.036	0.047
Tainan City	0.842	0.063	0.299	0.019	0.069
Kaohsiung City	**1.323**	0.066	**0.459**	0.022	0.124
Pingtung County	0.482	0.087	0.188	0.036	0.071
Taitung County	0.468	0.072	0.179	0.004	0.003
Hualien County	0.590	0.063	0.216	0.006	0.005
Yilan County	0.524	0.106	0.208	0.018	0.019

[#]Top five values in each column are bold.

Table S2. The probability of having 1000 infections given different numbers of initial infections in different cities (contact model). Colocation matrices in regular days were used. R_0=2.4.

	1	2	3	4	5	6	7	8	9	10
**Keelung City**	0.708	0.897	0.967	0.992	0.995	1.000	1.000	1.000	1.000	1.000
**New Taipei City**	0.750	0.942	0.982	0.998	1.000	1.000	1.000	1.000	1.000	1.000
**Taipei City**	0.773	0.935	0.990	0.998	1.000	1.000	1.000	1.000	1.000	1.000
**Taoyuan City**	0.665	0.907	0.962	0.985	0.996	0.999	1.000	1.000	1.000	1.000
**Hsinchu County**	0.539	0.776	0.887	0.948	0.978	0.992	0.995	1.000	1.000	1.000
**Hsinchu City**	0.669	0.897	0.960	0.988	0.995	0.998	0.999	1.000	1.000	1.000
**Miaoli County**	0.429	0.647	0.810	0.878	0.926	0.958	0.974	0.980	0.990	1.000
**Taichung City**	0.648	0.881	0.960	0.989	0.999	0.997	1.000	1.000	1.000	1.000
**Changhua County**	0.437	0.665	0.792	0.863	0.924	0.969	0.980	0.990	0.990	0.990
**Yunlin County**	0.354	0.541	0.731	0.780	0.884	0.930	0.952	0.980	0.980	0.990
**Chiayi County**	0.299	0.522	0.672	0.767	0.835	0.887	0.924	0.960	0.970	0.980
**Chiayi City**	0.645	0.841	0.948	0.978	0.990	0.995	0.998	1.000	1.000	1.000
**Nantou County**	0.359	0.641	0.792	0.871	0.914	0.956	0.961	0.990	0.990	0.990
**Tainan City**	0.600	0.848	0.946	0.972	0.993	0.995	1.000	1.000	1.000	1.000
**Kaohsiung City**	0.743	0.912	0.968	0.997	1.000	0.999	1.000	1.000	1.000	1.000
**Pingtung County**	0.479	0.734	0.850	0.927	0.966	0.977	0.983	1.000	0.990	1.000
**Taitung County**	0.438	0.702	0.812	0.910	0.944	0.958	0.982	0.990	0.990	1.000
**Hualien County**	0.480	0.753	0.872	0.934	0.971	0.980	0.988	1.000	1.000	1.000
**Yilan County**	0.459	0.704	0.853	0.894	0.955	0.972	0.976	1.000	0.990	1.000

Table S3. The probability of having 1000 infections given different numbers of initial infections in different cities (residence model). Movement data on weekdays were used. R_0=2.4.

	1	2	3	4	5	6	7	8	9	10
**Keelung City**	0.571	0.820	0.942	0.962	0.988	0.997	0.994	0.998	0.999	1.000
**New Taipei City**	0.593	0.835	0.923	0.968	0.987	0.992	1.000	1.000	1.000	1.000
**Taipei City**	0.646	0.842	0.926	0.973	0.989	0.996	0.998	0.999	1.000	0.999
**Taoyuan City**	0.614	0.826	0.922	0.965	0.988	0.995	0.999	0.999	1.000	1.000
**Hsinchu County**	0.587	0.824	0.933	0.956	0.984	0.996	0.997	1.000	1.000	1.000
**Hsinchu City**	0.615	0.821	0.910	0.975	0.993	0.997	1.000	1.000	1.000	1.000
**Miaoli County**	0.612	0.824	0.932	0.967	0.989	0.994	0.996	1.000	0.999	1.000
**Taichung City**	0.594	0.807	0.923	0.975	0.987	0.993	0.996	0.998	1.000	1.000
**Changhua County**	0.590	0.841	0.925	0.970	0.993	0.996	0.999	0.999	1.000	1.000
**Yunlin County**	0.583	0.833	0.938	0.964	0.987	0.998	0.996	1.000	1.000	1.000
**Chiayi County**	0.589	0.804	0.924	0.964	0.988	0.996	0.998	1.000	1.000	0.999
**Chiayi City**	0.568	0.848	0.937	0.967	0.993	0.995	0.997	1.000	1.000	1.000
**Nantou County**	0.587	0.810	0.927	0.975	0.982	0.993	1.000	0.999	1.000	1.000
**Tainan City**	0.563	0.830	0.934	0.969	0.987	0.993	0.998	0.999	0.999	1.000
**Kaohsiung City**	0.561	0.834	0.913	0.975	0.987	0.996	0.998	0.999	1.000	1.000
**Pingtung County**	0.565	0.844	0.939	0.969	0.989	0.995	1.000	1.000	1.000	1.000
**Taitung County**	0.616	0.843	0.922	0.964	0.982	0.993	0.998	1.000	1.000	1.000
**Hualien County**	0.574	0.836	0.922	0.955	0.992	0.994	0.998	0.999	1.000	1.000
**Yilan County**	0.586	0.823	0.928	0.979	0.985	0.997	0.997	0.999	1.000	1.000

Table S4. Proportion of each city's population that is a Facebook user

City name	Coverage
Yunlin County	0.13
Taitung County	0.13
Pingtung County	0.13
Chiayi County	0.14
Nantou County	0.14
Changhua County	0.14
Keelung City	0.14
Chiayi City	0.15
Kaohsiung City	0.15
Hualien County	0.15
Miaoli County	0.15
Taipei City	0.15
Yilan County	0.16
Tainan City	0.16
New Taipei City	0.17
Taichung City	0.17
Hsinchu City	0.18
Taoyuan City	0.18
Hsinchu County	0.19
